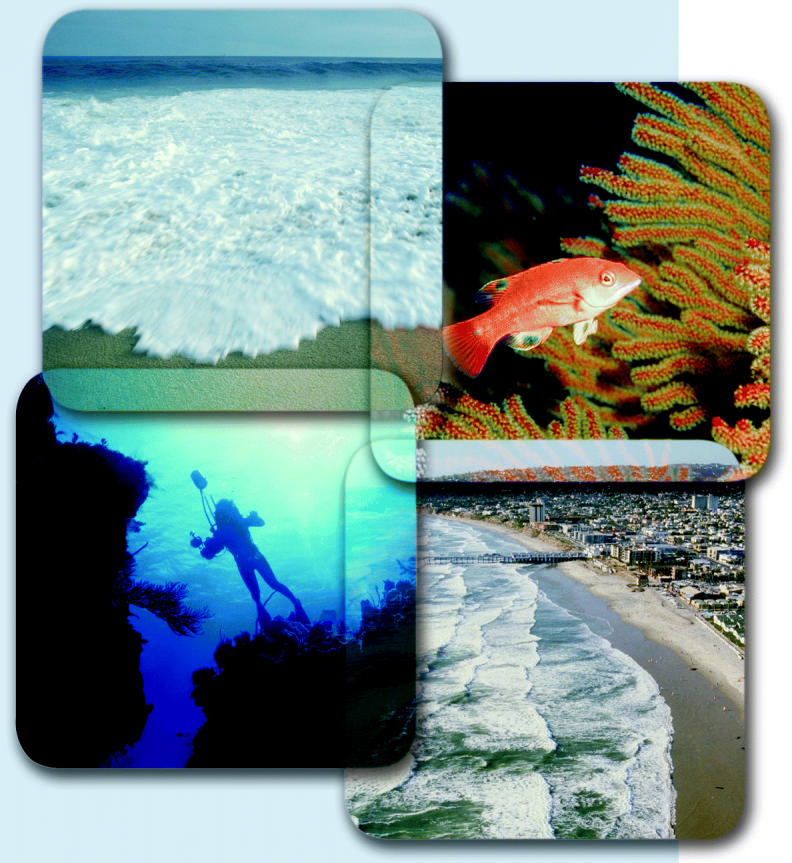# Centers for Oceans and Human Health

**Published:** 2005-02

**Authors:** Frederick L. Tyson

**Affiliations:** tyson2@niehs.nih.gov

The NIEHS and the National Science Foundation (NSF) have collaboratively launched four new Centers for Oceans and Human Health and in doing so have established a new paradigm for linking the health and rich resources of the Earth’s oceans with the health outcomes of the Earth’s population.

This program offers tremendous promise for developing more comprehensive linkages between oceans and human health as the world’s population continues to depend on one of our greatest natural resources for food, commerce, transportation, and recreation. It harnesses the various talents, disciplines, and expertise of scientists supported by the collaborating agencies. It combines the tools of genomics, proteomics, and metabolomics with physical oceanography. It also stimulates intercenter cooperation and coordination. Supported by the physical and biological science resources of the NSF and the NIEHS, the centers also demonstrate the capacity of federal research agencies to collaborate and leverage resources to foster high-quality interdisciplinary research.

This novel, groundbreaking interagency effort fulfills one of the key recommendations of the 2004 report *An Ocean Blueprint for the 21st Century: Final Report of the U.S. Commission on Ocean Policy*—to encourage interdisciplinary marine biomedical research to improve our understanding of the links between oceans and human health. [For more information on *An Ocean Blueprint*, see “America’s Oceans: A Blueprint for the Future,” p. A106 this issue.] In coming years, these NIEHS–NSF Centers for Oceans and Human Health will serve as models of the interaction between the life and physical sciences as well as of federal coordination. The program further serves as a model for interagency cooperation and the development of additional programs by federal agencies to address the links between oceans and human health.

The four centers are located at the University of Washington, the University of Hawaii, Woods Hole Oceanographic Institute, and the University of Miami. These centers are using oceanographic chemistry, genomics, proteomics, risk prevention, and public health approaches to address three specific areas in oceans and human health research: harmful algal blooms, water- and vectorborne pathogens, and the derivation of marine biopharmaceuticals.

The first annual meeting of the center directors was held in January 2005 and hosted by the University of Miami. The theme for this meeting was the exploration of opportunities for intercenter collaboration, leveraging, and synergy, as well as the pursuit of potential collaborative opportunities within initiatives sponsored by the Environmental Protection Agency and the National Oceanographic and Atmospheric Administration.

## Figures and Tables

**Figure:**